# Behavioral Factors Associated with Medication Nonadherence in Patients with Hypertension

**DOI:** 10.3390/ijerph18189614

**Published:** 2021-09-12

**Authors:** Shu-Mei Chang, I-Cheng Lu, Yi-Chun Chen, Chin-Feng Hsuan, Yin-Jin Lin, Hung-Yi Chuang

**Affiliations:** 1 Outpatient Department, E-DA Dachang Hospital, Kaohsiung 80794, Taiwan; ed109773@edah.org.tw (S.-M.C.); ed106481@edah.org.tw (Y.-J.L.); 2 Department of Occupational Medicine, E-DA Hospital, Kaohsiung 82445, Taiwan; ed102431@edah.org.tw; 3 School of Chinese Medicine for Post Baccalaureate, College of Medicine, I-Shou University, Kaohsiung 82445, Taiwan; 4 Department of Health Management, College of Medicine, I-Shou University, Kaohsiung 82445, Taiwan; 5 Department of Cardiology, E-DA Hospital, Kaohsiung 82445, Taiwan; Calvin.hsuan@msa.hinet.net; 6 School of Medicine, College of Medicine, I-Shou University, Kaohsiung 82445, Taiwan; 7 Department of Public Health, Kaohsiung Medical University, Kaohsiung 80708, Taiwan; ericch@kmu.edu.tw; 8 Department of Environmental and Occupational Medicine, Kaohsiung Medical University Hospital, Kaohsiung 80708, Taiwan

**Keywords:** hypertension, medication adherence, patient compliance, comorbidities

## Abstract

Medication nonadherence is associated with an increased risk of complications in hypertensive patients. We investigated behavioral factors associated with medication nonadherence in hypertensive patients in southern Taiwan. Using questionnaires, we collected data regarding clinicodemographic characteristics and nonadherence behaviors from 238 hypertensive patients. We assessed the self-reported prevalence of specific behaviors of medication nonadherence and investigated factors associated with each behavior using multivariable logistic regression analysis. The most common behavior of medication nonadherence was forgetting to take medication (28.6%), followed by discontinuing medication (9.2%) and reducing the medication dose (8.8%). Age ≥ 65 years (adjusted odds ratio [aOR] = 0.32, 95% confidence interval [CI] = 0.15–0.69) and male sex (aOR = 2.61, CI = 1.31–5.19) were associated with forgetting to take medication. The presence of comorbidities (diabetes, kidney disease, or both) and insomnia (aOR = 3.97, 95% CI = 1.30–12.1) was associated with reducing the medication dose. The use of diet supplements was associated with discontinuing the medication (aOR = 4.82, 95% CI = 1.50–15.5). Compliance with a low oil/sugar/sodium diet was a protective factor against discontinuing medication (aOR = 0.14; 95% CI = 0.03–0.75). The most pervasive behavior associated with medication nonadherence among hypertensive patients was forgetting to take medication. Age <65 years, male sex, comorbidities, insomnia, noncompliance with diet, and the use of dietary supplements were specifically associated with medication nonadherence.

## 1. Introduction

Hypertension is the main risk factor for cardiovascular disease, stroke, diabetes, and kidney disease worldwide [1], and its attributable burden of disease is greater than that of other risk factors, such as smoking and obesity [2]. It is a preventable disease that leads to significant disability and premature death [3], and adherence to its treatment protocol is essential to decrease the risk of complications, such as cardiovascular disease, cerebrovascular disease, diabetes, chronic kidney disease, and retinopathy [4,5,6]. However, medication nonadherence in patients with hypertension is a major public health issue. In this regard, a survey including 24 million patients with hypertension in the United States has shown that 31% were nonadherent to medication [7]. Furthermore, a meta-analysis of 28 studies from 15 countries reported that 45.2% of hypertensive patients and 31.2% of hypertensive patients with comorbidities did not comply with their antihypertensive drug regimen [8]. In Taiwan, poor medication adherence has been reported in approximately 15% of hypertensive patients and has been associated with an elevated risk of cardiovascular disease and all-cause hospitalizations [9].

Medication nonadherence among patients with hypertension has been associated with various factors [8,10,11,12,13]. Specifically, female sex, young age, number of drugs taken, smoking, the use of complementary and alternative medicine, and the presence of comorbidities have all been associated with an increased risk of noncompliance [8,10,11,13], whereas low salt intake and adequate exercise have been proposed as protective factors [12]. However, the association between demographic characteristics or lifestyle habits and medication nonadherence has not been consistent in previous studies, probably due to differences in ethnicity, culture, and health literacy among the populations studied, as well as the use of disparate measurement methods, such as pill counting and the Morisky medication adherence scale-8 [2,14,15,16]. In addition, it is unknown whether specific factors are related to particular behaviors, such as forgetting to take the medication, reducing the medication dose, or discontinuing the medication.

This study therefore aimed to investigate factors associated with nonadherence behaviors in relation to antihypertensive medication (forgetting to take the medication, reducing the medication dose, and discontinuing the medication). Additionally, we compared the differences in medication nonadherence between hypertensive patients with comorbidities (diabetes, kidney disease, or both) and hypertensive patients without comorbidities.

## 2. Materials and Methods

### 2.1. Study Participants

This cross-sectional study included patients aged 40 years or older diagnosed with hypertension with or without comorbidities (diabetes, kidney diseases, or both) and under antihypertensive medication for at least one year at the time of the assessment. Between January and December 2020, a trained and experienced interviewer approached patients at the waiting areas of three hospitals located in southern Taiwan and invited them to answer structured questionnaires if they fit the inclusion criteria. Two hundred and seventy-nine invited patients participated in this study; however, 41 patients were rejected during the interview. Overall, 141 hypertensive patients without comorbidities and 97 hypertensive patients with comorbidities were interviewed. This study was conducted in line with the principles of the Declaration of Helsinki and was approved by the Research Ethics Committee of E-Da Hospital (approval No. EMRP-108-065). The interviews were conducted with the understanding and the consent of each participant.

### 2.2. Data Collection

Ad hoc structured questionnaires were designed to collect information on demographic variables (age, sex, education, marital status), lifestyle habits (exercise, smoking, alcohol consumption, compliance with a low oil/sugar/sodium diet, use of dietary supplements), sleep condition, physical/mental health awareness, medication status (number of drugs taken and number of medication doses per day), and medication nonadherence. Medication nonadherence was evaluated by asking participants if the following behaviors occurred more than twice a week within the previous 3 months (with answers as “Yes” or “No”): Forgetting to take medication, reducing the medication dose, and discontinuing the medication. This questionnaire was self-developed according to previous literature [11,12,13,14,15] and clinical experiences, and the content validity was checked by five experts in the field to assess the usability, clarity, relevance, and comprehensibility of the content and questions (Figure S1).

### 2.3. Data Analysis

We calculated the frequencies of demographic characteristics, physical/mental health awareness, medication status, and medication nonadherence by univariate analysis, and compared the differences between participants with and without comorbidities using the chi-square test. The association between medication nonadherence and demographic variables, lifestyle habits, sleeping time, physical/mental health awareness, and medication status was assessed using the chi-square test. We used multivariable logistic regression analysis to investigate the association between specific behaviors of nonadherence and various factors; we considered age, sex, education level, marital status, presence of comorbidities, sleep condition, physical/mental health awareness, compliance with a low oil/sugar/sodium diet, and the use of dietary supplements as the independent variables.

**Figure 1:**
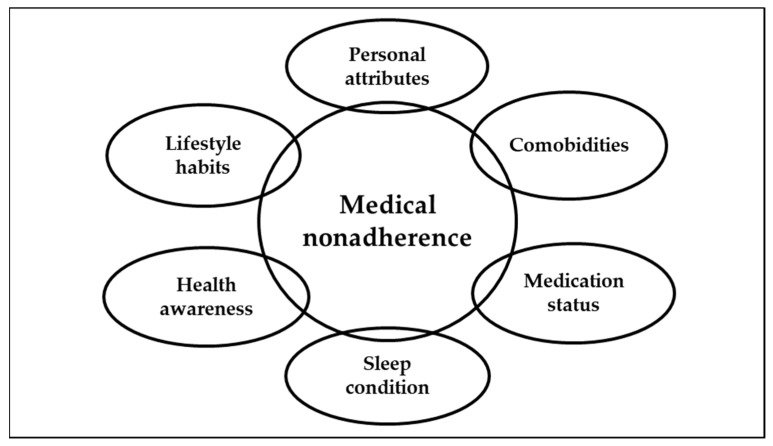
The considered factors associated with medical nonadherence.

The odds ratios (ORs) and 95% confidence intervals (CIs) were calculated and presented. The absence of comorbidities, age <65 years, female sex, education <9 years, married status, sleeping ≥7 h a day, good physical/mental health awareness, noncompliance with low oil/sugar/sodium diet, and no dietary supplementation were used as references in the multivariable logistic regression analysis. Data analysis was performed using SPSS software version 18 (IBM Corp., Armonk, NY, USA); the α value was set at 0.05. The conceptual framework is shown in Figure 1.

## 3. Results

### 3.1. Characteristics of the Included Hypertensive Patients

Of the 238 patients in this study, 54.6% were male, 44.3% were aged 65 years or older, and 73.8% were married. Additionally, 38.2% took more than three pills a day, and 66.4% took medication once a day (Table 1). Patients with comorbidities were more likely than patients without comorbidities to take more than three pills a day and to take medication more than once a day (54.2% vs. 28.7% and 43.3% vs. 27.0%, respectively; *p* < 0.05 for both comparisons). Forgetting to take medication (28.6%) was the most frequent behavior of medication nonadherence.

### 3.2. Factors Associated with Behaviors of Nonadherence

Table 2 shows the frequency of behaviors of nonadherence according to different variables. Age <65 years, male sex, marital status other than married, and alcohol consumption were associated with forgetting to take medication. Reduced sleeping time and multiple daily doses were associated with reducing the medication dose.

Table 3 shows the results of multivariable logistic regression analysis to identify factors associated with behaviors of medication nonadherence. Age ≥65 years constituted a protective factor for forgetting to take medication (adjusted OR [aOR] = 0.32, 95% CI = 0.15–0.69); however, male sex (aOR = 2.61, 95% CI = 1.31–5.19) was associated with forgetting to take medication. Additionally, the presence of comorbidities (aOR = 3.97, 95% CI = 1.30–12.1) was associated with reducing the medication dose. Reducing the medication dose was also associated with sleeping <7 h a day (aOR = 4.68, 95% CI = 1.42–15.4) and insomnia (aOR = 7.89, 95% CI = 1.22–51.3). Good compliance with a low oil/sugar/sodium diet was associated with a lower risk of discontinuing medication (aOR = 0.14, 95% CI = 0.03–0.75).

In contrast, the use of dietary supplements was also associated with discontinuing medication (aOR = 4.82, 95% CI = 1.50–15.5). The different protective and risk factors for behaviors of medication nonadherence are summarized in Table 4.

## 4. Discussion

This study investigated the association between specific behaviors related to medication nonadherence and different demographic, lifestyle, and clinical variables among hypertensive patients in Taiwan. Overall, the major medication noncompliant behavior exhibited by participants was forgetting to take medication. Age <65 years, male sex, presence of comorbidities, insomnia, and use of dietary supplements were risk factors for different nonadherence behaviors. Adequate compliance with a low oil/sugar/sodium diet was a protective factor for discontinuing the medication.

In this study, we found that <65 years of age was related to forgetting to take medication, which was consistent with the results of previous studies [2,12,13]. We speculate that younger patients are more prone to nonadherence due to a high work burden and a lack of an appropriate sense of illness. Previous research was conflicting regarding sex-related differences in antihypertensive medication compliance, with some studies showing a higher risk of nonadherence in women [2,8,13] and others showing no difference between the sexes [12,14]. However, our findings showed that men were more likely to forget taking medication. Lin et al. [17] found that compared to female patients, male patients with diabetes were associated with poorer medication adherence, and another study found that the male patients in Taiwan had lower adherence to daily food guides [18]. Additionally, measurements of medication nonadherence in previous studies consisted of pill counts or scores in scales, which are different from our assessments. Further studies are required to elucidate sex-related differences in medication adherence.

Despite the fact that diabetes, kidney disease, or both were the sole comorbidities assessed in this study, we found that these were associated with a higher risk of reducing the medication dose. As expected, hypertensive patients with comorbidities took a higher number of drugs and required multiple doses more frequently than hypertensive patients without comorbidities. A study analyzing the health system data of Midwestern Americans found that the number of comorbidities present and the number of drugs taken were directly associated with poorer medication adherence [13]. Gupta et al. [2] came to a similar conclusion using drug metabolite assessments as indicators of medication nonadherence in British and Czech populations.

In this study, sleep disorders were associated with medication nonadherence; insomnia, in particular, significantly increased the likelihood of reducing the medication dose. This is consistent with the results of previous studies [19,20]. Additionally, we found that using dietary supplements was associated with a 4.82-fold increase in the risk of discontinuing medication, compared with not using dietary supplements, which is in line with the results from previous studies showing that hypertensive patients who use complementary and alternative therapies have low adherence to antihypertensive medication [11,15]. Furthermore, our study found that adequate compliance with a low oil/sugar/sodium diet was associated with good medication adherence. The link between a high salt diet and poor antihypertensive medication compliance has been described in previous studies [12,21], and Abu et al. [22] showed that patients with little knowledge of hypertension are likely to not restrict their salt intake. These findings suggest that interventions directed at increasing health literacy and encouraging lifestyle modifications could be useful in improving medication adherence in hypertensive patients.

To the best of our knowledge, few studies have investigated the factors associated with specific behaviors of medication nonadherence. Our study provides valuable information that may help medical staff accurately design education programs and interventions to improve the adherence of hypertensive patients to medication. However, this study has several limitations. Since data were collected through interviews and consisted of self-reports, social-desirability bias and recall bias are a concern. Besides, the health literacy of hypertensive patients may have been a confounding factor in this study. Additionally, as diabetes, kidney disease, or both are the sole comorbidities assessed in this study, the medical nonadherence of hypertensive patients with other comorbidities needs to be evaluated. Finally, since the characteristics of patients from hospitals in southern Taiwan may differ from those of patients from other hospitals or clinics, our results may not be generalizable to other districts.

Previous research has shown that adherence to antihypertensive medications is directly associated with a high quality of care, but not with the amount of time the medical staff spends with the patients [23]. Therefore, we believe that, in order to prevent nonadherence with antihypertensive treatment, healthcare providers should improve their communicative skills, implement tailored education programs, and generate specific interventions aimed at increasing commitment to therapy, especially in young patients with multiple comorbidities. Further investigations are required to find the impact of communication with hypertensive patients in their medication adherence.

## 5. Conclusions

In this study, the most common medication nonadherence behavior among patients with hypertension was forgetting to take medication. Age <65 years, male sex, the presence of comorbidities, insomnia, and the use of dietary supplements were risk factors for nonadherence to antihypertensive medication, whereas compliance with a low oil/sugar/sodium diet was associated with a lower risk of discontinuing medication.

## Figures and Tables

**Table 1:** Baseline characteristics and behaviors of nonadherence in the total cohort and in patients with or without comorbidities *.

Variables	Comorbidities *		*p*-Value	Total
	No	Yes		
	*n* (%)	*n* (%)		*n* (%)
Age, years (*n* = 235)			0.233	
<65	82 (59.0)	49 (51.0)		131 (55.7)
≥65	57 (54.8)	47 (49.0)		104 (44.3)
Sex (*n* = 238)			0.895	
Female	63 (44.7)	45 (46.4)		108 (45.4)
Male	78 (55.3)	52 (53.6)		130 (54.6)
Education, years (*n* = 237)			0.407	
<9	50 (35.5)	37 (38.5)		87 (36.7)
10–12	45 (31.9)	23 (24.0)		68 (28.7)
≤12	46 (32.6)	36 (37.5)		82 (34.6)
Marital status (*n* = 237)			0.881	
Married	104 (74.3)	71 (73.2)		175 (73.8)
Other	36 (25.7)	26 (26.8)		62 (26.1)
Physical health awareness (*n* = 238)			0.219	
Normal/good	121 (85.8)	89 (91.8)		210 (88.2)
Poor	20 (14.2)	8 (8.2)		28 (11.8)
Mental health awareness (*n* = 238)			0.869	
Normal/good	114 (80.9)	77 (79.3)		191 (80.3)
Poor	27 (19.1)	20 (20.6)		47 (19.7)
Sleep condition (*n* = 237)			0.355	
>7 h a day	72 (51.1)	58 (60.4)		130 (54.9)
<7 h a day	61 (43.3)	33 (34.4)		94 (39.7)
Insomnia	8 (5.7)	5 (5.2)		13 (5.5)
Exercise (*n* = 238)			0.889	
No	46 (32.6)	33 (34.0)		79 (33.2)
Yes	95 (67.4)	64 (66.0)		159 (66.8)
Smoking (*n* = 238)			0.573	
No	119 (84.4)	85 (87.6)		204 (85.7)
Yes	22 (15.6)	12 (12.4)		34 (14.3)
Alcohol consumption (*n* = 238)			0.763	
No	105 (74.5)	74 (76.3)		179 (75.2)
Often/occasionally	36 (25.5)	23 (23.7)		59 (24.8)
Compliance with low oil/sugar/sodium diet (*n* = 238)			0.113	
Never	10 (7.1)	15 (15.5)		25 (10.5)
Often	60 (42.6)	36 (37.1)		96 (40.3)
Always	71 (50.4)	46 (47.4)		117 (49.2)
Use of dietary supplements (*n* = 237)			1.00	
No	68 (48.2)	47 (49.0)		115 (48.5)
Yes	73 (51.8)	49 (51.0)		122 (51.5)
Number of drugs (*n* = 232)			<0.001	
<3	97 (71.3)	44 (45.8)		141 (59.2)
>3	39 (28.7)	52 (54.2)		91 (38.2)
Number of Medication doses per day (*n* = 238)			0.012	
1	103 (73.0)	55 (56.7)		158 (66.4)
>1	38 (27.0)	42 (43.3)		80 (33.6)
Forgetting to take medication (*n* = 238)			0.058	
No	94 (66.7)	76 (78.4)		170 (71.4)
Yes	47 (33.3)	21 (21.6)		68 (28.6)
Reducing the dose (*n* = 236)			0.061	
No	131 (94.2)	84 (86.6)		215 (90.3)
Yes	8 (5.8)	13 (13.4)		21 (8.8)
Discontinuing medication (*n* = 238)			0.255	
No	125 (88.7)	91 (93.8)		216 (90.8)
Yes	16 (11.3)	6 (6.2)		22 (9.2)

* Diabetes, kidney disease, or both.

**Table 2:** Distribution of behaviors of nonadherence to medication according to demographic variables, lifestyle habits, sleep condition, physical/mental health awareness, and medication status.

Variables	Medication Nonadherence Behaviors *					
	Forgetting to Take Medication		Reducing Medication Dose		Discontinuing Medication	
	No	Yes	No	Yes	No	Yes
Age, years						
<65	80 (61.1)	51 (38.9)	121 (93.1)	9 (6.9)	117 (89.3)	14 (10.7)
≥65	89 (85.6)	15 (14.4)	92 (89.3)	11 (10.7)	98 (94.2)	6 (5.8)
*p*-value	<0.001		0.351		0.240	
Sex						
Female	90 (83.3)	18 (16.7)	99 (92.5)	8 (7.5)	99 (91.7)	9 (8.3)
Male	80 (61.5)	50 (38.5)	116 (89.9)	13 (10.1)	117 (90.0)	13 (10.0)
*p*-value	<0.001		0.647		0.823	
Education, years						
≤9	69 (79.3)	18 (20.7)	76 (88.4)	10 (11.6)	80 (92.0)	7 (8.0)
10–12	43 (63.2)	25 (36.8)	63 (94.0)	4 (6.0)	64 (94.1)	4 (5.9)
>12	57 (69.5)	25 (30.5)	76 (92.7)	6 (7.3)	71 (86.6)	11 (13.4)
*p*-value	0.081		0.411		0.252	
Marital status						
Married	134 (76.6)	41 (23.4)	154 (88.5)	20 (11.5)	157 (89.7)	18 (10.3)
Other	36 (58.1)	26 (41.9)	61 (98.4)	1 (1.6)	58 (93.5)	4 (6.5)
*p*-value	0.008		0.018		0.454	
Physical health awareness						
Normal/Good	149 (71.0)	61 (29.0)	192 (91.9)	17 (8.1)	192 (91.4)	18 (8.6)
Poor	21 (75.0)	7 (25.0)	23 (85.2)	4 (14.8)	24 (85.7)	4 (14.3)
*p*-value	0.824		0.275		0.305	
Mental health awareness						
Normal/Good	136 (71.2)	55 (28.8)	174 (92.1)	15 (7.9)	176 (92.1)	15 (7.9)
Poor	34 (72.3)	13 (27.7)	41 (87.2)	6 (12.8)	40 (18.5)	7 (14.9)
*p*-value	1.00		0.388		0.159	
Sleeping time						
>7 h a day	90 (69.2)	40 (30.8)	124 (96.1)	5 (3.9)	120 (92.3)	10 (7.7)
<7 h a day	69 (73.4)	25 (26.6)	80 (86.0)	13 (14.0)	84 (89.4)	10 (10.6)
Insomnia	10 (76.9)	3 (23.1)	10 (76.9)	3 (23.1)	11 (84.6)	2 (15.4)
*p*-value	0.713		0.006		0.557	
Exercise						
No	53 (67.1)	26 (32.9)	72 (92.3)	6 (7.7)	70 (88.6)	9 (11.4)
Yes	117 (73.6)	42 (26.4)	143 (90.5)	15 (9.5)	146 (91.8)	13 (8.2)
*p*-value	0.361		0.809		0.478	
Smoking						
No	150 (73.5)	54 (26.5)	181 (89.6)	21 (10.4)	187 (91.7)	17 (8.3)
Yes	20 (58.8)	14 (41.2)	34 (100.0)	0 (0.0)	29 (85.3)	5 (14.7)
*p*-value	0.100		0.051		0.216	
Alcohol						
No	136 (76.0)	43 (24.0)	162 (91.5)	15 (8.5)	166 (92.7)	13 (7.3)
Yes	34 (57.6)	25 (42.4)	53 (89.8)	6 (10.2)	50 (84.7)	9 (15.3)
*p*-value	0.012		0.792		0.074	
Compliance with low oil/sugar/sodium diet						
Never	17 (68.0)	8 (32.0)	24 (96.0)	1 (4.0)	21 (84.0)	4 (16.0)
Often	65 (67.7)	31 (32.3)	85 (90.4)	9 (9.6)	87 (90.6)	9 (9.4)
Always	88 (75.2)	29 (24.8)	106 (90.6)	11 (9.4)	108 (92.3)	9 (7.7)
*p*-value	0.446		0.660		0.428	
Use of dietary supplements						
No	82 (71.3)	33 (28.7)	107 (93.9)	7 (6.1)	108 (93.9)	7 (6.1)
Yes	88 (72.1)	34 (27.9)	107 (88.4)	14 (11.6)	107 (87.7)	15 (12.3)
*p*-value	1.00		0.173		0.119	
Number of drugs						
≤3	97 (68.8)	44 (31.2)	129 (91.5)	12 (8.5)	124 (87.9)	17 (12.1)
>3	70 (76.9)	21 (23.1)	81 (91.0)	8 (9.0)	86 (94.5)	5 (5.5)
*p*-value	0.231		1.00		0.112	
Number of Medication doses per day						
1	112 (70.9)	46 (29.1)	148 (94.3)	9 (5.7)	146 (92.4)	12 (7.6)
>1	58 (72.5)	22 (27.5)	67 (84.8)	12 (15.2)	70 (87.5)	10 (12.5)
*p*-value	0.880		0.027		0.240	

* Data are shown as n (%). All comparisons were performed using the chi-square test.

**Table 3:** Multivariable logistic regression analysis of factors associated with specific behaviors of nonadherence to antihypertensive medication.

Variables	Medication Nonadherence Behaviors		
	Forgetting to Take Medication	Reducing Medication Dose	Discontinuing Medication
	OR (95% CI)	OR (95% CI)	OR (95% CI)
Comorbidities ^a^			
No	1.0	1.0	1.0
Yes	0.57 (0.29–1.12)	3.97 (1.30–12.1) *	0.38 (0.12–1.24)
Age, years			
<65	1.0	1.0	1.0
≥65	0.32 (0.15–0.69)*	1.24 (0.38–4.07)	0.58 (0.17–2.01)
Sex			
Female	1.0	1.0	1.0
Male	2.61 (1.31–5.19)*	2.36 (0.77–7.25)	1.22 (0.42–3.52)
Education, years			
<9	1.0	1.0	1.0
10–12	1.31 (0.56–3.09)	0.54 (0.13–2.24)	0.43 (0.09–2.04)
>12	1.00 (0.42–2.36)	0.64 (0.16–2.52)	1.76 (0.50–6.25)
Marital status			
Married	1.0	1.0	1.0
Other	1.90 (0.93–3.86)	0.13 (0.02–1.06)	0.27 (0.06–1.27)
Physical health awareness			
Normal/Good	1.0	1.0	1.0
Poor	1.14 (0.35–3.66)	1.31 (0.24–7.08)	1.12 (0.22–5.80)
Mental health awareness			
Normal/Good	1.0	1.0	1.0
Poor	0.64 (0.25–1.64)	1.23 (0.32–4.80)	1.17 (0.29–4.71)
Compliance with low oil/sugar/sodium diet			
Never	1.0	1.0	1.0
Often	1.00 (0.34–2.94)	1.49 (0.14–16.5)	0.23 (0.04–1.20)
Always	0.83 (0.28–2.45)	1.70 (0.16–18.5)	0.14 (0.03–0.75)*
Use of dietary supplements			
No	1.0	1.0	1.0
Yes	1.22 (0.64–2.33)	2.86 (0.90–9.11)	4.82 (1.50–15.5)*
Sleeping time			
>7 h	1.0	1.0	1.0
<7 h	0.90 (0.46–1.79)	4.68 (1.42–15.4)*	1.47 (0.51–4.30)
Insomnia	0.83 (0.18–3.84)	7.89 (1.22–51.3)*	1.98 (0.28–13.7)

^a^ diabetes, kidney disease, or both. * *p* < 0.05. OR, odds ratio. CI, confidence interval.

**Table 4:** Summary of factors associated with specific behaviors of nonadherence to antihypertensive medication in this study, according to the multivariable logistic regression analysis.

	Medication Nonadherence Behaviors		
	Forgetting to Take Medication	Reducing Medication Dose	Discontinuing Medication
Risk factors	Age <65 years	Comorbidities (diabetes, kidney disease, or both)	Dietary supplements
	Male sex	Insomnia	
Protective factor			Compliance with low oil/sugar/sodium diet

## Data Availability

Not applicable.

## Supplementary Materials

The following are available online at https://www.mdpi.com/article/10.3390/ijerph18189614/s1, Figure S1: The questionnaire.
