# Supplementary material for: Behavioral Factors Associated with Medication Nonadherence in Patients with Hypertension
Source: Int J Environ Res Public Health. 2021 Sep 12;18(18):9614. doi: 10.3390/ijerph18189614 (PMC8469687; doi:10.3390/ijerph18189614)
Supplement: Supplementary file 1 [file ijerph-18-09614-s001.zip › ijerph-1306030-supplementary.pdf]

Figure S1. Questionnaire

Title: Behavioral factors associated with medication nonadherence in patients with hypertension

Visit: _____ year _____ month _____ day No.:________________

Interviewer:________________

Hello.

We are the outpatient nurse and case manager of Eda Dachang Hospital, Kaohsiung. We are currently conducting a study on the factors related to medical nonadherence in hypertensive patients. With your consent, your personal information will only be used for this study, and the information will be strictly protected from leakage and governed by the ethics of the study.

Shu-May Chang,
Nurse of outpatient
Eda Dachang Hospital Clinic

(A) Basic Information

Name:__________________________ Chart number:________________________

Sex: □ Male □ female

Birthday: _____ year _____ month _____ day

Marital status: □ married □ others

Education years: □ <9 □ 10-12 □ >12

Do you have a job now? □ No □ Yes

(B) Life style

- Do you currently have a habit of smoking? (smoking habit: refers to smoking at least once a day for more than half a year) □Yes □ no
- Have you ever drunk alcohol? □Often □ occasionally □ no
- Do you have a habit of exercising regularly? (Regular exercise habits (defined as 3

times): Refers to exercise at least 3 times a week, at least 30 minutes each time, and lasts for more than half a year. Exercises include walking, brisk walking, etc.)

□Yes □ no

- Do you have use of dietary supplements for more than half a year? □Yes □ no
- Do you currently have a habit of drinking tea? (Tea drinking habits: drink at least once a week for more than half a year; 1 cup is about 200c.c) □ no □ 1-2 cups a week □ 3-6 cups a week □ everyday
- Do you currently have a habit of drinking coffee? (Coffee drinking habits: drink at least once a week for more than half a year; 1 cup is about 200c.c) □ no □ 1-2 cups a week □ 3-6 cups a week □ everyday
- Do you compliant with low oil/sugar/sodium diet? □ never □ often □ always
- What is your current sleeping condition: □>7 hours a day □ <7 hours a day □ insomnia

(C) Personal health condition

- Have you ever been diagnosed by a physician with any of the following diseases (past medical history): ____
- Generally speaking, how do you feel about your physical health compared to people of the same age: □ good □ normal □ poor
- Generally speaking, how do you feel about your mental health compared to people of the same age: □ good □ normal □ poor

(D) Medication adherence

- Have you ever forgotten to take your medication more than twice a week within the previous 3 months? □Yes □ no
- Have you ever reduced the medication dose more than twice a week within the previous 3 months? □Yes □ no

- Have you ever discontinued the medication more than twice a week within the previous 3 months? □Yes □ no

(E) Drug use

- What kinds of medications do you currently take for your disease? ____________
- How many pills did you take in total? ____________________
- How many times a day do you take your medication?_________________
